# Characteristics of the gut microbiota of patients with symptomatic carotid atherosclerotic plaques positive for bacterial genetic material

**DOI:** 10.3389/fcimb.2023.1296554

**Published:** 2024-01-12

**Authors:** Hang Lv, Zhiyuan Zhang, Bo Fu, Zhongchen Li, Tengkun Yin, Chao Liu, Bin Xu, Dawei Wang, Baojie Li, Jiheng Hao, Liyong Zhang, Jiyue Wang

**Affiliations:** ^1^ School of Clinical Medicine, Shandong Second Medical University, Weifang, Shandong, China; ^2^ Department of Neurosurgery, Liaocheng People’s Hospital, Liaocheng, Shandong, China; ^3^ Department of Precision Medicine, Liaocheng People’s Hospital, Liaocheng, Shandong, China; ^4^ Department of Orthopedics, Liaocheng People’s Hospital, Liaocheng, Shandong, China; ^5^ Bio-X Research Institute, Shanghai Jiao Tong University, Shanghai, China

**Keywords:** carotid atherosclerosis, gut microbiota, plaque, inflammation, microbial function

## Abstract

**Background:**

The gut microbiota (GM) is believed to be closely associated with symptomatic carotid atherosclerosis (SCAS), yet more evidence is needed to substantiate the significant role of GM in SCAS. This study, based on the detection of bacterial DNA in carotid plaques, explores the characteristics of GM in SCAS patients with plaque bacterial genetic material positivity, aiming to provide a reference for subsequent research.

**Methods:**

We enrolled 27 healthy individuals (NHF group) and 23 SCAS patients (PFBS group). We utilized 16S rDNA V3-V4 region gene sequencing to analyze the microbiota in fecal samples from both groups, as well as in plaque samples from the carotid bifurcation extending to the origin of the internal carotid artery in all patients.

**Results:**

Our results indicate significant differences in the gut microbiota (GM) between SCAS patients and healthy individuals. The detection rate of bacterial DNA in plaque samples was approximately 26%. Compared to patients with negative plaques (PRSOPWNP group), those with positive plaques (PRSOPWPP group) exhibited significant alterations in their GM, particularly an upregulation of 11 bacterial genera (such as Klebsiella and Streptococcus) in the gut, which were also present in the plaques. In terms of microbial gene function prediction, pathways such as Fluorobenzoate degradation were significantly upregulated in the GM of patients with positive plaques.

**Conclusion:**

In summary, our study is the first to identify significant alterations in the gut microbiota of patients with positive plaques, providing crucial microbial evidence for further exploration of the pathogenesis of SCAS.

## 1 Introduction

Stroke is currently the second most common cause of death worldwide and the primary cause of disability in adults (Feigin et al., 2022). Carotid atherosclerosis (CAS) plaque formation, leading to carotid stenosis, rupture and detachment of unstable plaques, and local thrombosis, are key contributors to ischemic stroke (IS), significantly impacting clinical prognosis. Patients with symptomatic carotid atherosclerosis (SCAS) typically present with mild cerebral ischemic symptoms, such as minor ischemic stroke, transient ischemic attacks, or temporary blindness, and have a higher risk of recurrent cardiovascular and cerebrovascular events compared to those with asymptomatic carotid atherosclerosis, often requiring close monitoring and active treatment (Thapar et al., 2013; Wabnitz and Turan, 2017). Factors like male gender, smoking, alcohol consumption, dysregulated glucose and lipid metabolism, and inflammatory states are significant risk factors influencing CAS development and plaque stability (Willerson, 2002; Song et al., 2020). Therefore, the prevention and treatment of SCAS have become urgent health issues needing resolution.

The human gut microbiota (GM) constitutes a vast “ecosystem,” with bacteria comprising over 99% of its makeup, closely associated with human health and disease. Recent studies have identified a close relationship between GM and carotid atherosclerosis (CAS). On one hand, significant changes in GM have been observed in SCAS patients, potentially promoting the development of SCAS by influencing the body’s inflammatory regulatory pathways (Karlsson et al., 2012). On the other hand, DNA from various bacteria originating from the oral cavity and gut, as well as some live bacteria, have been detected in CAS plaques, potentially exacerbating local inflammation within the plaques (Kozarov et al., 2005; Koren et al., 2011).

The stability of atherosclerotic plaques is often associated with multiple factors such as local inflammation, endothelial dysfunction, and angiogenesis (Willerson, 2002; Ylä-Herttuala et al., 2013). Increasing evidence suggests that bacteria within plaques might have the potential to affect plaque stability. Previous studies have shown a positive correlation between bacterial DNA in plaques and the number of inflammatory cells present (Koren et al., 2011), and can activate Toll-like receptors in macrophages and endothelial cells within the plaques (El-Zayat et al., 2019), inducing processes like foam cell formation and endothelial cell damage (Jin et al., 2023). Additionally, live bacteria in plaques, such as Porphyromonas gingivalis, have the ability to translocate through oral epithelium into the bloodstream, adhere to, and invade vascular endothelial cells (Farrugia et al., 2021), inducing endothelial dysfunction, promoting foam cell formation, proliferation and calcification of vascular smooth muscle cells, as well as angiogenesis within the plaque (Zhang et al., 2021). Biofilms enhance bacterial resistance, and bacteria have been reported to exist as biofilm deposits in plaques. Hormonal levels in the body can promote the dispersion of biofilms, increasing the risk of plaque rupture (Lanter et al., 2014).

In this study, we investigated the microbiota in fecal and plaque samples from SCAS patients, focusing particularly on the GM of patients with positive bacterial genetic material in plaques. This provides foundational theoretical evidence for subsequent exploration of prevention and treatment methods for SCAS patients in Northern China.

## 2 Methods

### 2.1 Study population

We consecutively enrolled 23 SCAS patients (2 females, 21 males) who underwent carotid endarterectomy (CEA) at the Neurosurgery Department of Liaocheng People’s Hospital from April 2020 to May 2021, along with 27 healthy individuals (12 females, 15 males) confirmed through health examinations at the same hospital’s medical examination center during the same period. The diagnosis of all patients was primarily confirmed by digital subtraction angiography (DSA) of the whole brain MRS and assisted by carotid ultrasound. Basic information and clinical data of all participants were collected through interviews and laboratory tests. Written informed consent was obtained from all participants, and the study was approved by the Ethics Committee of Liaocheng People’s Hospital (Approval Number: 2021120).

Inclusion criteria for patients were as follows: (1) age greater than 18 years; (2) presence of atherosclerotic plaque formation in the left, right, or bilateral carotid arteries (defined as intima-media thickness >1.4 mm or focal wall thickening at least 50% greater than the surrounding vessel wall); (3) presence of mild ischemic stroke symptoms, such as episodic headaches, transient ischemic attacks, or temporary visual disturbances; (4) patients diagnosed with carotid atherosclerotic stenosis by DSA examination and requiring surgical treatment.

Exclusion criteria included: (1) concomitant inflammatory bowel disease and a history of gastrointestinal surgery in the past 3 months; (2) severe coagulation disorders; (3) poorly controlled diabetes, with blood glucose levels exceeding 300 mg/dl; (4) use of antibiotics, probiotics, prebiotics, or gastrointestinal medications within the past 3 months; (5) the presence of severe cardiovascular, pulmonary, hepatic, renal, hematologic, endocrine, or neoplastic diseases; (6) pregnancy or the perinatal period.

### 2.2 Data collection and specimen collection

All participant information was collected through face-to-face interviews by attending physicians at our hospital, including age, gender, and medical history. Digital Subtraction Angiography (DSA) via the femoral artery route was used to examine and confirm the cerebral vascular status of all patients. Blood samples from all participants were collected and analyzed after fasting for 10 hours, with tests including blood cell count, blood glucose, and lipid metabolism indicators. After standard collection of fecal samples from all participants, they were immediately frozen and stored at -80°C. Plaques excised from the carotid bifurcation to the origin of the internal carotid artery during CEA in all patients were immediately frozen and stored in liquid nitrogen tanks for future use.

### 2.3 Sample DNA extraction, DNA library construction, sequencing, and operational taxonomic unit analysis

Microbial DNA extraction from fecal and plaque samples was performed using the TIANamp Micro DNA Kit (TIANGEN, Beijing, China). The V3-V4 region of 16S rDNA has been chosen as the target interval for PCR amplification, utilizing 341F (CCTAYGGGRBGCASCAG) and 806R (GGACTACNNGGGTATCTAAT) as primers (Rintala et al., 2017). Subsequently, sequencing libraries were prepared using the TRUSEQ^®^ DNA PCR Sample Preparation Kit (Illumina, USA). The quality of the prepared libraries was assessed using a Qubit^®^ 2.0 Fluorometer (Thermo Scientific) and an Agilent Bioanalyzer 2100 system. High-quality libraries were subjected to sequencing using the Illumina HiSeq 2500 platform (CapitalBio Technology Co., Ltd., Beijing, China). Sequence data were then clustered into operational taxonomic units (OTUs) based on a 97% similarity threshold using Usearch (Version 11.0.667). Taxonomic analysis of representative OTU sequences at the 97% similarity level was performed against the Silva database (Release 132). The QIIME software was employed to generate microbial abundance information at various taxonomic levels.

### 2.4 Statistical analysis

Statistical analyses were performed using R version 3.6.0 and SPSS version 27. Various statistical methods, including Student’s t-test, Wilcoxon rank-sum test, Tukey’s test, Kruskal-Wallis test, chi-square test, and Linear Discriminant Analysis, were utilized to analyze clinical data, microbial abundance data, and microbial functional data. Continuous variables were presented as means ± standard deviations. Prior to analysis, normality was assessed, with a P-value of ≥0.05 indicating normal distribution. Subsequent parametric or non-parametric tests were conducted, with a P-value of <0.05 indicating statistical significance. Categorical variables were represented numerically, and chi-square tests were employed for difference testing, with a P-value of <0.05 indicating statistical significance. R version was employed for microbial community diversity analysis, differential significance analysis, and Spearman correlation analysis.

## 3 Results

### 3.1 Demographic and clinical characteristics of the subjects

We conducted a statistical analysis of the clinical data (**Supplementary Table 1**) of the participants and found significant differences in smoking and drinking between the 23 patients (PFBS group) and the 27 healthy adults (NHF group). In terms of risk factors, the PFBS group had significantly higher levels of Lp(a), WBC, FBG, TyG, N, NLR, and SUA compared to the healthy control group. Conversely, levels of HDL-C, ApoA-I, and L were lower in the PFBS group, with no significant differences in the remaining indicators (**Table 1**).

**Table 1:** Clinical characteristics of patients and healthy subjects.

Features	PFBS (n=23)	NHF (n=27)	P value
Gender	Male:21, Female:2	Male:15, Female:12	0.013*
Age	65.17 ± 5.39	62.22 ± 7.41	0.119
Smoking	Yes:18, No:5	Yes:11, No:16	0.007*
Drinking	Yes:20, No:3	Yes:10, No:17	0.001*
TC	4.11 ± 1.01	4.41 ± 0.71	0.224
TG	0.92 ± 0.34	0.81 ± 0.48	0.336
HDL-C	1.15(0.94,1.30)	1.27(1.14,1.43)	0.018*
LDL-C	2.40 ± 0.81	2.74 ± 0.79	0.143
VLDL-C	0.22(0.16,0.29)	0.21(0.16,0.27)	0.579
Lp (a)	325.70 ± 224.59	145.74 ± 90.10	0.001*
ApoA-I	1.07 ± 0.15	1.51 ± 0.30	<0.001*
ApoE	39.82 ± 13.83	39.99 ± 5.92	0.956
ApoB	0.83 ± 0.27	0.92 ± 0.20	0.189
FFA	0.50(0.44,0.60)	0.54(0.36,0.72)	0.392
WBC	7.83 ± 2.43	6.47 ± 1.66	0.03*
FBG	6.31(5.44,7.99)	4.96(4.38,5.49)	<0.001*
TyG	5.93(4.03,7.81)	4.03(1.71,6.14)	0.01*
N	4.78(3.41,7.70)	3.99(2.66,5.07)	0.019*
L	1.58 ± 0.54	2.04 ± 0.59	0.006*
NLR	3.12(2.44,5.57)	2.04(1.24,2.38)	0.001*
SUA	273.00(250.00,356.00)	213.00(143.00,297.00)	0.05*

* denotes statistically significant differences as determined by Student’s T-test or Wilcoxon rank-sum test.

Subsequently, based on the detection of bacterial DNA in plaque samples, the 23 patient samples were divided into a positive plaque group (PP group, 6 cases) and a negative plaque group (17 cases). Further, based on the plaque results, the patients’ fecal samples were categorized into a positive plaque patient group (6 cases, PRSOPWPP group) and a negative plaque patient group (17 cases, PRSOPWNP group). There were no significant differences in lifestyle habits (smoking, drinking) between the two groups. However, in terms of laboratory data, the positive plaque patients exhibited significantly higher levels of ApoE, WBC, FBG, N, and NLR compared to the negative plaque patients, with no significant differences in the other indicators (**Table 2**).

**Table 2:** Clinical characteristics of patients with positive and negative plaques.

Features	PRSOPWPP(n=6)	PRSOPWNP (n=17)	P value
Gender	Male:6,Female:0	Male:15,Female:2	1
Age	65.83 ± 5.67	64.94 ± 5.45	0.736
Smoking	Yes:5,No:1	Yes:13,No:4	1
Drinking	Yes:6,No:0	Yes:14,No:3	0.539
TC	4.12 ± 1.57	4.10 ± 0.79	0.988
TG	1.06 ± 0.30	0.87 ± 0.35	0.259
HDL-C	1.15(0.93,1.27)	1.15(0.92,1.31)	0.806
LDL-C	2.50 ± 1.35	2.37 ± 0.58	0.135
VLDL-C	0.22(0.17,0.25)	0.24(0.15,0.32)	0.599
Lp (a)	394.17 ± 227.95	301.53 ± 225.26	0.398
ApoA-I	1.03 ± 0.17	1.09 ± 0.14	0.398
ApoE	56.77 ± 11.17	33.84 ± 8.85	<0.001*
ApoB	0.87 ± 0.43	0.82 ± 0.19	0.829
FFA	0.57 ± 0.09	0.46 ± 0.15	0.112
WBC	9.76(8.57,11.22)	5.97(5.57,8.68)	0.013*
FBG	8.38(6.36,9.66)	5.56(5.04,6.89)	0.027*
TyG	6.93(6.21,11.9)	4.88(3.38,7.45)	0.052
N	7.49(6.13,9.02)	3.88(3.35,5.69)	0.008*
L	1.39 ± 0.56	1.65 ± 0.54	0.325
NLR	4.89(3.91,10.35)	2.64(2.08,3.67)	0.014*
SUA	255(225.25,330.00)	281(252.50,362.50)	0.362

TC, Total Cholesterol; TG, Triglycerides; HDL-C, High-Density Lipoprotein Cholesterol; LDL-C, Low-Density Lipoprotein Cholesterol; VLDL-C, Very Low-Density Lipoprotein Cholesterol; Lp (a), Lipoprotein (a); ApoA-I, Apolipoprotein AI; ApoE, Apolipoprotein E; ApoB, Apolipoprotein B; FFA, Free Fatty Acids; WBC, White Blood Cells; FBG, Fasting Blood Glucose; TyG, Triglycer-ide-Glucose Index; N, Neutrophils; L, Lymphocytes; NLR, Neutrophil-Lymphocyte Ratio; SUA, Serum Uric Acid. Values are presented as mean ± SD or median (interquartile range). * denotes statistically significant differences as determined by Student’s T-test or Wilcoxon rank-sum test.

### 3.2 Community diversity and statistical analysis

#### 3.2.1 Species composition analysis

**Figure 1:**
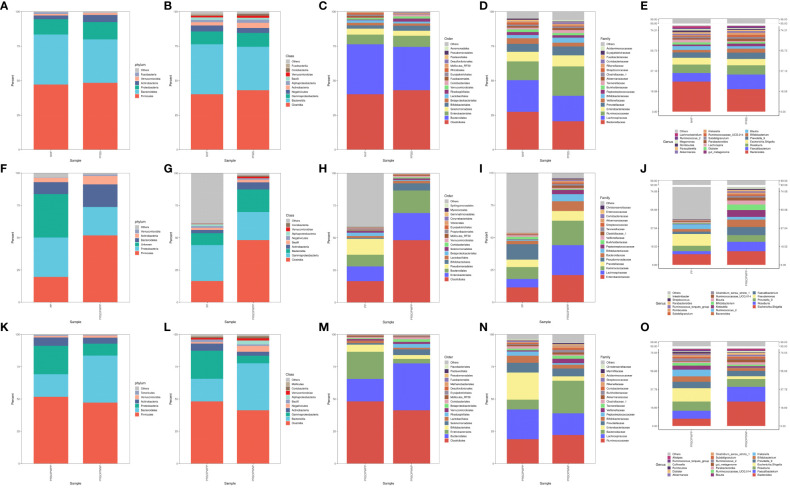
Bar charts of species composition at the phylum, class, order, family, and genus levels. **(A-E)** Composition and abundance of the core microbiota in the NHF and PFBS groups. **(F-J)** Composition and abundance of the core microbiota in the PP and PRSOPWPP groups. **(K-O)** Composition and abundance of the core microbiota in the PRSOPWPP and PRSOPWNP groups.

Bacterial DNA was detected in 6 of the 23 SCAS patient plaque samples, yielding a positivity rate of approximately 26%. We identified 15 bacterial phyla, 24 classes, 43 orders, 78 families, and 224 genera common to both the PP and PRSOPWPP groups. We created bar charts of the species composition analysis for all participant sample groups at five taxonomic levels: phylum, class, order, family, and genus (**Supplementary Tables 2, 3**). These charts visually represent and compare the microbial community composition between the NHF and PFBS groups (**Figures 1A–E**), between the PP and PRSOPWPP groups (**Figures 1F–J**), and between the PRSOPWPP and PRSOPWNP groups (**Figures 1K–O**).

#### 3.2.2 Alpha diversity and beta diversity analysis of microbiota

**Figure 2:**
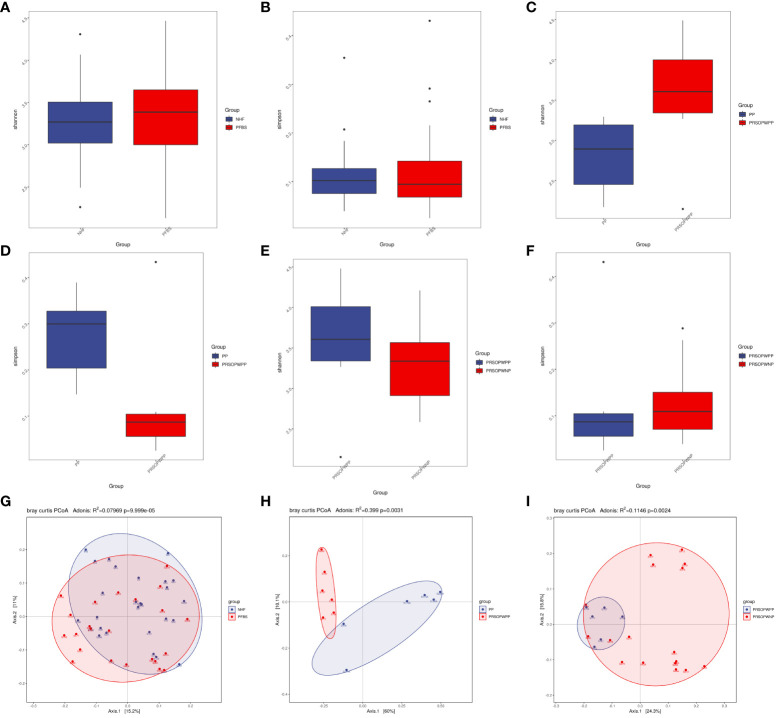
Comparison of α-diversity and β-diversity of microbiota. **(A-F)** Comparison of α-diversity as determined by Shannon and Simpson indices between the NHF and PFBS groups, PP and PRSOPWPP groups, and PRSOPWPP and PRSOPWNP groups. **(G-I)** Comparison of β-diversity as determined by Principal Coordinate Analysis (PCoA) between the NHF and PFBS groups, PP and PRSOPWPP groups, and PRSOPWPP and PRSOPWNP groups.

Statistical tests such as the T-test for the Shannon index (P<0.05 indicating significant difference) and the Wilcoxon rank-sum test for the Simpson index (P<0.05 indicating significant difference) can be used to reflect the statistical differences in species α-diversity between groups. Our results show no statistical difference in species α-diversity between the NHF and PFBS groups (Shannon index, P= 0.47, **Figure 2A**; Simpson index, P= 0.92, **Figure 2B**), between the PP and PRSOPWPP groups (Shannon index, P= 0.09, **Figure 2C**; Simpson index, P= 0.06, **Figure 2D**), and between the PRSOPWPP and PRSOPWNP groups (Shannon index, P= 0.57, **Figure 2E**; Simpson index, P= 0.47, **Figure 2F**).

Principal Coordinates Analysis (PCoA; Adonis multivariate analysis of variance, where R² indicates the explanatory power of the grouping for sample differences, P<0.05 indicating significant difference) results can reflect similarities and differences in species β-diversity. Our results indicate significant statistical differences in microbial community β-diversity between the NHF and PFBS groups (Adonis, R²=0.08, P<0.05; **Figure 2G**), between the PP and PRSOPWPP groups (Adonis, R²=0.40, P<0.05; **Figure 2H**), and between the PRSOPWPP and PRSOPWNP groups (Adonis, R²=0.11, P<0.05; **Figure 2I**).

#### 3.2.3 Significance analysis of microbial community differences

Our primary focus was on the differences in microbial communities between the NHF and PFBS groups, and between the PRSOPWPP and PRSOPWNP groups.

**Figure 3:**
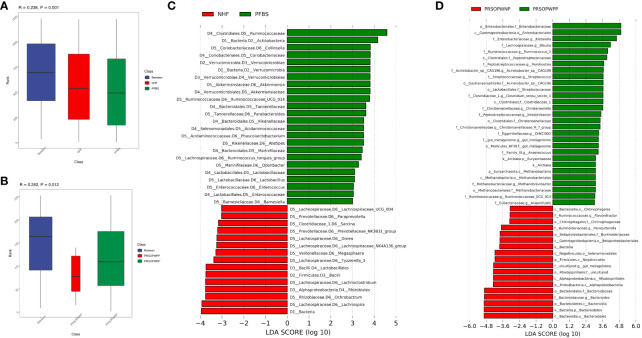
Significant analysis of differences in gut microbiota between groups. **(A, B)** Anosim analysis (analysis of similarity) for NHF and PFBS groups, and PRSOPWPP and PRSOPWNP groups. This non-parametric test, with p < 0.05, indicates meaningful group differentiation. **(C, D)** Identification of significantly different gut bacteria between NHF and PFBS groups, and PRSOPWPP and PRSOPWNP groups using LEfSe (Linear Discriminant Analysis Effect Size). An LDA score > 3 and p < 0.05 denote significant differences. LEfSe is used to assess the effect size of linear discriminant analysis.

Firstly, we employed Analysis of Molecular Variance (AMOVA, P<0.05 indicating significant difference) to test for significant differences between the microbial communities of the groups. Results showed significant differences between the NHF and PFBS groups (P<0.05), and between the PRSOPWPP and PRSOPWNP groups (P<0.05). Secondly, Anosim analysis (R> 0 indicating greater inter-group than intra-group differences, P<0.05 indicating significant difference) and Multi Response Permutation Procedure (MRPP) analysis (A> 0 indicating greater inter-group than intra-group differences, P<0.05 indicating significant difference) were used to determine whether the differences between the groups were greater than within the groups, thus validating the significance of the grouping. Our results indicated that both the NHF and PFBS groups (Anosim, R=0.24, P<0.05; MRPP, A=0.04, P<0.05; **Figure 3A**), and the PRSOPWPP and PRSOPWNP groups (Anosim, R=0.28, P<0.05; MRPP, A=0.07, P<0.05; **Figure 3B**) exhibited greater inter-group differences, confirming meaningful group distinctions. Finally, we employed Linear Discriminant Analysis Effect Size (LEfSe, LDA score >3, P<0.05 indicating significant difference) to identify taxa with significant differences in abundance between groups (Lin and Peddada, 2020). At the genus level, 22 differentially abundant bacteria (LDA score >3) were identified between the NHF and PFBS groups, with each group enriched in 11 different genera (**Figure 3C**). In the PRSOPWPP and PRSOPWNP groups, 2 phyla, 4 classes, 7 orders, 12 families, and 20 genera (LDA score >3) were identified as differentially abundant. Specifically, the PRSOPWPP group was enriched in 1 phylum, 1 class, 2 orders, 8 families (1 unknown), and 16 genera (1 unknown), while the PRSOPWNP group was enriched in 1 phylum, 3 classes, 5 orders, 4 families (1 unknown), and 4 genera (1 unknown) (**Figure 3D**) (**Supplementary Table 4**).

Specifically, we found that the GM of both the NHF and PFBS groups was primarily composed of four abundant phyla (Firmicutes, Bacteroidetes, Proteobacteria, Actinobacteria), with Verrucomicrobia and Actinobacteria significantly enriched in the PFBS group. The GM of patients in the PRSOPWPP and PRSOPWNP groups was similarly dominated by these four phyla. Notably, Bacteroidetes, enriched in the PRSOPWNP group, and Euryarchaeota, enriched in the PRSOPWPP group, exhibited significant differences between the two groups.

At the genus level, there were 22 genera with significant differences in abundance between the NHF and PFBS groups. Eleven genera (Collinsella, Akkermansia, Ruminococcaceae_UCG_014, Parabacteroides, Phascolarctobacterium, Alistipes, Ruminococcus_torques_group, Odoribacter, Lactobacillus, Enterococcus, Barnesiella) were significantly enriched in the PFBS group, while the remaining genera (Lachnospira, Ochrobactrum, Lachnoclostridium, Tyzzerella_3, Megasphaera, Lachnospiraceae_NK4A136_group, Dorea, Prevotellaceae_NK3B31_group, Sarcina, Paraprevotella, Lachnospiraceae_UCG_004) were significantly enriched in the NHF group.

At the genus level, Escherichia-Shigella had the highest abundance in the PRSOPWPP group, differing from the PRSOPWNP group (Bacteroides). Additionally, Acinetobacter_sp._CAG196, Anaerococcus, Anaerofustis, Blautia, CHKCI002, Christensenella, Christensenellaceae_R_7_group, Clostridium_sensu_stricto_1, Intestinibacter, Klebsiella, Methanobrevibacter, Romboutsia, Ruminococcaceae_UCG_013, Ruminococcus_2, Streptococcus, and an unknown genus belonging to Mollicutes_RF39 were significantly enriched in the PRSOPWPP group; while Bacteroides, Flavonifractor, Parasutterella, and an unknown genus belonging to Rhodospirillales, enriched in the PRSOPWNP group, showed significant differences between the groups.

Furthermore, we discovered that 11 genera significantly enriched in the gut of patients with positive plaques were present in the plaques, namely Streptococcus, Blautia, Klebsiella, Clostridium_sensu_stricto_1, Romboutsia, Ruminococcaceae_UCG-013, Ruminococcus_2, Intestinibacter, Christensenellaceae_R-7_group, Anaerococcus, and Methanobrevibacter.

### 3.3 Association between gut microbiota and clinical characteristics

**Figure 4:**
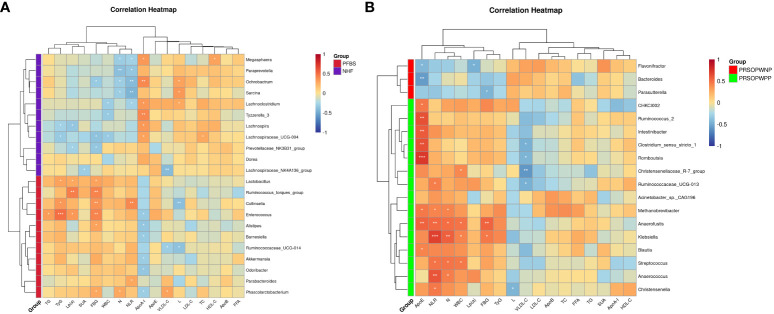
Correlation between gut microbiota and clinical characteristics. **(A)** Correlation between significantly different bacterial genera and clinical characteristics between NHF and PFBS groups, and **(B)** between PRSOPWPP and PRSOPWNP groups.

We employed Spearman correlation analysis to explore the intrinsic connections between significantly different GM and clinical features. The results revealed that among the 11 bacterial genera significantly enriched in the PFBS group’s gut, 6 were positively correlated with FBG levels, 6 negatively correlated with ApoA-I levels, 3 positively correlated with TyG levels, 3 positively correlated with Lp(a) levels, 2 positively correlated with NLR levels, 2 negatively correlated with L levels, 1 positively correlated with N levels, 1 positively correlated with TG levels, and 1 positively correlated with VLDL-C levels. (**Figure 4A**).

Furthermore, we found that the 15 known genera significantly enriched in the PRSOPWPP group had close relationships with clinical features. Specifically, 8 bacteria were positively correlated with ApoE levels, 7 with NLR levels, 5 with N levels, 4 with WBC levels, and 2 with FBG levels. Additionally, 4 bacteria were negatively correlated with VLDL-C levels, and 1 with L levels. (**Figure 4B**).

### 3.4 Functional profile of the microbiota

We used PICRUST 2 (Phylogenetic Investigation of Communities by Reconstruction of Unobserved States) software based on the Greengenes database to predict functional profiles of microbial genes. We conducted predictive analyses on Kyoto Encyclopedia of Genes and Genomes (KEGG) and Clusters of Orthologous Groups (COG) functional categories to observe differences and changes in functional gene expression related to metabolic pathways and protein functions in the microbial communities (**Supplementary Table 5**).

**Figure 5:**
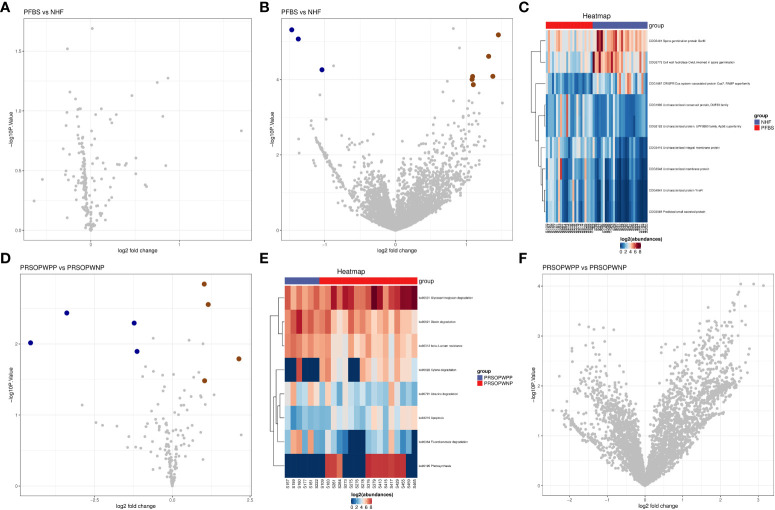
Predictive analysis of functional expression of gut microbiota. **(A)** Volcano plot of the differential analysis of KEGG metabolic pathways between NHF and PFBS groups. **(B, C)** Volcano plots of differential COG protein functions analysis between NHF and PFBS groups, and clustering heatmaps of significantly different protein functions. **(D, E)** Volcano plots of the differential analysis of KEGG metabolic pathways between PRSOPWPP and PRSOPWNP groups, and clustering heatmaps of significantly different metabolic pathways. **(F)** Volcano plot of the differential analysis of COG protein functions between PRSOPWPP and PRSOPWNP groups.

We found that there were no significant differences in the expression of predicted metabolic pathways between the gut microbiota of the NHF and PFBS groups (**Figure 5A**). However, there were significant differences in the expression of 9 predicted protein functions (**Figures 5B, C**). Compared to the NHF group, 6 predicted protein functions were significantly upregulated in the PFBS group, with a notable upregulation of COG1900, an enzyme involved in anaerobic homocysteine biosynthesis.

In the gut microbiota of the PRSOPWPP and PRSOPWNP groups, significant differences were observed in the expression of 8 predicted metabolic pathways (**Figures 5D, E**), but no significant differences were noted in the expression of predicted protein functions (**Figure 5F**). Compared to the PRSOPWNP group, the PRSOPWPP group showed significant upregulation in 4 predicted metabolic pathways: Fluorobenzoate degradation, Dioxin degradation, Atrazine degradation, and beta-Lactam resistance.

### 3.5 Correlation between functional expression of gut microbiota, clinical features, and differentially abundant bacterial genera

We employed Spearman correlation analysis to explore the potential connections between microbial functional expression, clinical indices, and differentially abundant bacterial genera.

**Figure 6:**
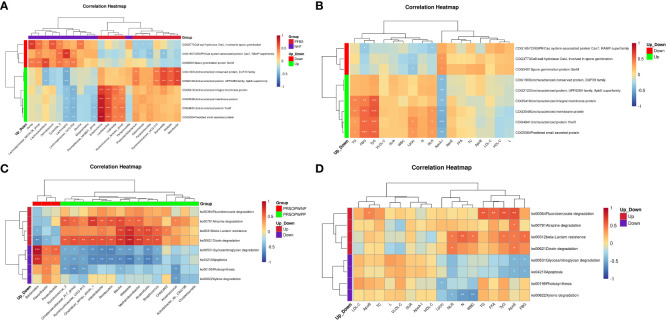
Correlation analysis between functional expression of gut microbiota, clinical characteristics, and different bacterial genera. **(A)** Correlation between significantly different bacterial genera and differential protein functions between NHF and PFBS groups. **(B)** Correlation between differential protein functions and clinical characteristics between NHF and PFBS groups. **(C)** Correlation between significantly different bacterial genera and differential metabolic pathways be-tween PRSOPWPP and PRSOPWNP groups. **(D)** Correlation between differential metabolic pathways and clinical characteristics between PRSOPWPP and PRSOPWNP groups.

The results indicated that in the PFBS group, the significantly enriched genera Enterococcus, Lactobacillus, Collinsella, and Ruminococcus_torques_group were positively correlated with the expression of COG4841, COG3548, COG5584, and COG5416, respectively. Phascolarctobacterium, Ruminococcaceae_UCG-014, Barnesiella, Alistipes, and Odoribacter were positively correlated with the expression of COG2122 and COG1900 (**Figure 6A**). Furthermore, all upregulated predicted protein functions in the PFBS group were negatively correlated with ApoA-I levels, and four predicted protein functions (COG4841, COG3548, COG5584, and COG5416) were positively correlated with levels of TG, TyG, and FBG (**Figure 6B**).

We also discovered that within the PRSOPWPP group, the Dioxin degradation pathway showed the most numerous positive correlations with significantly enriched bacterial species (n=11, p<0.05), followed by Atrazine degradation (n=10, p<0.05), and beta-Lactam resistance (n=7, p<0.05) (**Figure 6C**). Additionally, significantly upregulated predicted metabolic pathways were positively correlated with lipid indices, inflammatory markers, FBG, and TyG levels (**Figure 6D**).

## 4 Discussion

The primary objective of this study was to investigate the characteristics of the gut microbiota in patients with positive arterial plaques and their potential link to carotid artery plaques. We observed that compared to the NHF group, individuals in the PFBS group had more prevalent smoking and drinking habits. Clinical data indicated metabolic dysregulation in these patients, such as impaired glucose and lipid metabolism and inflammatory states, which might contribute to the development of carotid atherosclerosis and affect plaque stability (Tang et al., 2018; Sterpetti, 2020; Lubrano and Balzan, 2021). Furthermore, based on the detection of bacterial DNA in plaques, we compared clinical data between patients with positive and negative plaques, marking the first comparative study of these two groups in clinical aspects. The findings showed that patients with positive plaques had significantly higher levels of ApoE, FBG, WBC, N, and NLR. These indicators suggest more severe metabolic dysregulation and inflammatory states in patients with positive plaques, potentially exacerbating CAS progression and plaque instability.

Given the potential link between GM and CAS, we analyzed the GM of all participants. We found dysbiosis in the GM of SCAS patients, with certain bacteria like Collinsella significantly enriched in the gut, consistent with previous studies (Karlsson et al., 2012). We also analyzed the microbiota in SCAS patient plaques and found that some bacteria in the plaques were shared with the GM of patients with positive plaques, further confirming that at least some plaque bacteria may originate from the gut, in line with previous findings (Koren et al., 2011). As previously discussed, the bacteria in plaques may have a potential connection with plaque stability. The likelihood of bacterial translocation to the plaque and the amount translocated (i.e., the bacterial DNA content) often relate to the host’s physiological state, the characteristics of the plaque region, and the bacteria’s inherent properties (Pizarro-Cerdá and Cossart, 2006). We hypothesize that the GM of patients with positive plaques may differ from those with negative plaques, possibly related to more severe physiological abnormalities in the former and potentially linked to translocation of gut bacteria to the plaques.

We found significant differences in β-diversity of GM between patients with positive and negative plaques. Further analysis revealed 16 genera (1 unknown) significantly enriched in the gut of patients with positive plaques, indicating a marked difference in GM compared to those with negative plaques. Correlation analysis showed that most bacteria enriched in the gut of patients with positive plaques were significantly positively correlated with elevated clinical indicators, suggesting that changes in GM could be one of the factors exacerbating abnormal physiological states.

We conducted a predictive analysis of the gut microbiota’s functional expression in all participants to explore potential changes in the functionality of the gut microbiota (GM) and its potential associations with physiological states of the body. We discovered that the predicted expression of an enzyme involved in the biosynthesis of anaerobic homocysteine (COG1900) was significantly upregulated in the gut microbiota of SCAS patients. Previous research has shown that elevated homocysteine levels are independently associated with the morphology and increased area of carotid artery plaques (Alsulaimani et al., 2013), closely linked to plaque progression and vulnerability (Yang et al., 2014; Ben et al., 2020), and a subclinical marker for stroke risk (Zhang et al., 2020; Rabelo et al., 2022). This suggests that GM might indirectly promote the development of SCAS by upregulating the expression of COG1900. Furthermore, we found significant upregulation in specific predicted metabolic pathways in the gut microbiota of patients with positive plaques. Specifically, the Fluorobenzoate degradation pathway was most upregulated in patients with positive plaques. This metabolic pathway is also upregulated in inflammatory diseases such as osteoarthritis and Crohn’s disease (Gevers et al., 2014; Wang et al., 2021), indicating its potential involvement in the body’s inflammatory regulation. The aryl hydrocarbon receptor (Ahr) is associated with human inflammatory responses (Rothhammer and Quintana, 2019), and Dioxin, involved in activating Ahr (Furue et al., 2021), can attenuate inflammation through mechanisms like thymic atrophy, apoptosis, Treg induction, and induction of myeloid-derived suppressor cells upon activation by 2,3,7,8-tetrachlorodibenzo-p-dioxin (TCDD, one of the Dioxin compounds) (Cannon et al., 2021). Therefore, upregulation of the Dioxin degradation pathway might reduce Ahr activation, weakening its anti-inflammatory role in the body. The significant upregulation of Beta-Lactam resistance suggests that drug-resistant bacteria might predominate in the gut of patients with positive plaques, potentially triggering aberrant inflammatory responses of the immune system and affecting the body’s ability to resist infection (von Klitzing et al., 2017). In summary, these three upregulated predicted metabolic pathways may be associated with the more severe inflammatory state in patients with positive plaques. Clostridium, a potential Atrazine-degrading bacterium (Fang et al., 2015), belongs to the same family as Clostridium_sensu_stricto_1. In our results, Clostridium_sensu_stricto_1 was enriched in patients with positive plaques and showed the greatest positive correlation with the expression of the Atrazine degradation pathway. Therefore, we speculate it might be a potential Atrazine-degrading bacterium. Correlation analysis indicated that the 16 bacterial genera enriched in the gut of patients with positive plaques were significantly positively correlated with most of the upregulated metabolic pathways, suggesting these genera might participate in the expression of these metabolic pathways.

Our study found that 11 bacterial genera significantly enriched in the gut of patients with positive plaques were also present in the plaques themselves, suggesting that these bacteria might have the potential to translocate from the gut to the plaques. The integrity of the gut barrier, composed of the mucous layer, intestinal epithelial cells, and immune cells, is crucial for human survival, health, and defense (Farhadi et al., 2003; Vancamelbeke and Vermeire, 2017). Patients with positive plaques were mostly elderly, and aging is a key potential factor in gut barrier dysfunction (Man et al., 2014; Untersmayr et al., 2022). Coupled with these patients’ history of alcohol consumption, significant hyperglycemia, and systemic inflammation, these factors likely facilitate bacterial translocation from the gut to the bloodstream (Leclercq et al., 2014; Li et al., 2016; Thaiss et al., 2018). Macrophages and neutrophils in the blood can phagocytize bacteria that enter the bloodstream (Sharma et al., 2022), and immune cells carrying bacteria can migrate and accumulate in areas of carotid atherosclerosis under the influence of chemotactic factors (Gencer et al., 2021), potentially indirectly increasing the amount of bacterial DNA in the plaques.The characteristics of the bacteria themselves are also a crucial determinant of translocation. Some bacteria within the Klebsiella genus, such as Klebsiella pneumoniae, can translocate across intestinal epithelia via a cell invasion mechanism dependent on Rho GTPases and phosphatidylinositol 3-kinase/Akt (Wyres et al., 2020). Streptococcus and Klebsiella can adhere and colonize through adhesion molecules like pili and adhesins (Nobbs et al., 2009; Chen et al., 2023). Some species of Blautia have genes encoding phage and transposons, facilitating their adherence and colonization (Shen et al., 2020; Liu et al., 2021). Overgrowth of Clostridium_sensu_stricto_1 is associated with necrotizing enterocolitis (Yang et al., 2019), which may facilitate its translocation across intestinal epithelia (Ciftci et al., 2012). Species of Clostridium_sensu_stricto_1 are linked to various infectious diseases (Gubler et al., 1989; Tappe et al., 2009; Daganou et al., 2016), and there have been case reports of Romboutsia causing human marrow necrosis (Seviar et al., 2022), suggesting these genera may have certain adhesive and colonization capabilities (Boyle and Finlay, 2003). Mucins play a significant role in the gut barrier mechanism (Breugelmans et al., 2022), and Ruminococcus_2 is associated with their degradation (Hatayama et al., 2023), indicating that this genus might translocate by disrupting the integrity of the gut barrier. Anaerococcus, commonly residing in the skin and gastrointestinal tract, can cause infections and lead to bacteremia under certain conditions (Murphy and Frick, 2013; Badri et al., 2019; Cobo et al., 2021), indicating its adhesive and colonization capabilities. Additionally, evidence shows that Streptococcus (Domenech et al., 2012), Klebsiella (Alcántar-Curiel et al., 2013), and Methanobrevibacter (Bang et al., 2014) have the ability to form biofilms, suggesting strong survival capabilities. If they exist in plaques as biofilm deposits, they might increase plaque instability. The abnormal physiological state of patients with positive plaques might exacerbate pathological changes like endothelial dysfunction in CAS areas, facilitating the adherence and colonization of free bacteria (Lemichez et al., 2010).

Interestingly, there were no significant differences between Streptococcus, Klebsiella, Blautia, and Clostridium_sensu_stricto_1 in the NHF and PFBS groups, while significant differences were observed between patients with positive and negative plaques, and these genera were present in the plaques. Therefore, we speculate that these genera may not have the potential to cause CAS but may affect plaque stability.

This study has several limitations. First, our patient recruitment strategy, which involved continuous enrollment, did not adequately balance gender distribution. However, previous research indicates that GM variations are mainly due to strokes or TIAs caused by major artery atherosclerosis (Yin et al., 2015), similar to our study population. Therefore, the impact of gender on our GM results might be minor. Second, this is a single-center study with a small sample size. Future multi-center studies with larger sample sizes are needed to validate our findings. Lastly, we did not fully consider the potential impact of antidiabetic medications on GM, which will be addressed in future large-scale studies through subgroup analyses to mitigate such potential influences.

In summary, patients with positive plaques exhibit more severe metabolic disorders and inflammatory states, along with significant enrichment of bacteria in the gut, particularly those capable of translocating across the intestinal barrier, adhering, colonizing, and forming biofilms. These capabilities provide favorable conditions for their translocation to plaque regions and local infection, increasing the bacterial DNA content in the plaques. This may promote plaque instability and, consequently, increase the risk of ischemic stroke.

## 5 Conclusion

Our study demonstrates that patients with symptomatic carotid atherosclerosis (SCAS) exhibit metabolic disorders and inflammatory states, along with significant changes in their gut microbiota (GM), consistent with previous research findings. By analyzing bacterial DNA in plaques, we compared for the first time the clinical characteristics and GM differences between patients with negative and positive plaques. We found that patients with positive plaques have more severe physiological abnormalities, which may further damage the endothelial integrity in the carotid plaque regions (facilitating bacterial adhesion and colonization) and compromise plaque stability. Significant differences were observed in the GM between patients with positive and negative plaques, particularly the significant enrichment of 11 bacterial genera in the gut of patients with positive plaques, which were also present in the plaques. Some of these bacteria have the ability to translocate to the plaques, potentially exacerbating plaque instability through increased local infection, inflammation, and bacterial DNA content. In conclusion, these findings may enhance our understanding of plaque stability in SCAS and help identify patients at high risk of plaque instability. Future research should further explore the differing roles and specific molecular mechanisms of various microbes in plaque formation and evolution, as well as how GM modulation could intervene in the plaque stability of SCAS.

## Data availability statement

The datasets presented in this study can be found in online repositories. The names of the repository/repositories and accession number(s) can be found below: https://www.ncbi.nlm.nih.gov/, PRJNA1012713.

## Ethics statement

The studies involving humans were approved by Liaocheng People’s Hospital Ethics Committee. The studies were conducted in accordance with the local legislation and institutional requirements. The participants provided their written informed consent to participate in this study.

## Author contributions

HL: Conceptualization, Formal Analysis, Methodology, Project administration, Writing – original draft, Writing – review & editing. ZZ: Conceptualization, Data curation, Investigation, Supervision, Writing – original draft, Writing – review & editing. BF: Conceptualization, Formal Analysis, Investigation, Visualization, Writing – original draft, Writing – review & editing. ZL: Resources, Supervision, Writing – review & editing. TY: Investigation, Supervision, Writing – review & editing. CL: Investigation, Writing – review & editing. BX: Investigation, Writing – review & editing. DW: Writing – review & editing, Resources. BL: Writing – review & editing, Resources. JH: Funding acquisition, Project administration, Writing – review & editing. LZ: Funding acquisition, Project administration, Writing – review & editing. JW: Funding acquisition, Methodology, Project administration, Writing – review & editing.

## Supplementary material

The Supplementary Material for this article can be found online at: https://www.frontiersin.org/articles/10.3389/fcimb.2023.1296554/full#supplementary-material

Supplementary Tables 1–5 Details of the participants and experimental results.
